# Pyroptosis in myocardial ischemia-reperfusion injury: mechanistic pathways and therapeutic prospects

**DOI:** 10.3389/fcell.2026.1938791

**Published:** 2026-10-13

**Authors:** Shenshen Ding, Xiaoding Zhang, Zuxian Sheng, Yuanyuan Wang, Wuwang Fang

**Affiliations:** 1 Department of Cardiology, Second Affiliated Hospital of Nanjing University of Chinese Medicine, Nanjing, China; 2 Department of General Practice, Xi’an Daxing Hospital, Xian, China

**Keywords:** cardiac cells, inflammatory response, myocardial ischemia-reperfusion injury, NLRP3 inflammasome, pyroptosis, therapeutic strategies

## Abstract

Acute myocardial infarction (AMI) remains the leading cause of cardiovascular death and disability worldwide. With the advances in percutaneous coronary intervention and coronary artery bypass grafting, timely revascularization has become the main approach to limit infarct size and improve heart function and prognosis. However, the sudden restoration of blood flow during reperfusion can actually accelerate heart muscle cell death by worsening inflammation, calcium overload, and oxidative stress, a phenomenon known as myocardial ischemia-reperfusion injury (MIRI). Pyroptosis, a lytic and highly pro-inflammatory form of programmed cell death executed by members of the gasdermin family, has emerged as a central mechanism linking cell death to inflammation in MIRI. This review summarizes the molecular pathways of pyroptosis, including the canonical NLRP3/caspase-1/GSDMD axis, non-canonical caspase-4/5/11 activation, and the caspase-3/GSDME pathway, and profiles the roles of individual gasdermin family members in MIRI. We then delineate how pyroptosis operates in cardiomyocytes, cardiac fibroblasts, vascular endothelial cells, and macrophages; how it is modulated by oxidative stress, calcium overload, and autophagy. We further evaluate therapeutic agents acting at distinct nodes of this cascade, including NLRP3 inhibitors, caspase inhibitors, GSDMD inhibitors, repurposed drugs, and natural products, and assess the experimental models and levels of evidence supporting them. We conclude that pyroptosis is a promising therapeutic target for MIRI. Nevertheless, the available evidence is still dominated by preclinical studies of GSDMD, and several issues remain unresolved, including cell type-specific regulatory mechanisms, the roles of gasdermin family members beyond GSDMD, and key translational challenges.

## Introduction

Cardiovascular disease (CVD) continues to exact a staggering global death toll (Woodruff et al., 2024). Among these conditions, acute myocardial infarction (AMI) persists as a dominant contributor to both morbidity and mortality (Gerczuk and Kloner, 2012). In most instances, AMI most commonly arises from the rupture of an unstable atherosclerotic plaque or erosion of the coronary endothelium, and is accordingly classified as type 1 myocardial infarction. Plaque rupture releases abundant thrombogenic material, which triggers mural thrombus formation and occludes the coronary artery. Alternatively, atherosclerotic debris may detach and embolize the distal vasculature. The consequence is myocardial ischemia, which often progresses to frank necrosis (Reed et al., 2017). Beyond type 1, the spectrum encompasses type 2 (oxygen supply–demand mismatch), type 3 (fatal MI in which death precedes biomarker confirmation), type 4a (PCI-related), type 4 b (stent thrombosis), type 4c (in-stent restenosis), and type 5 (CABG-related) myocardial infarction. Timely reperfusion—whether achieved by thrombolysis, primary percutaneous coronary intervention (PPCI), or coronary artery bypass grafting—remains the single most effective strategy to limit infarct size, preserve cardiac function, and improve clinical outcomes (Thygesen et al., 2012). Reperfusion clears the occluded vessel and restores flow; however, the act of reperfusion itself can inflict additional damage, including cardiomyocyte death. This paradox is termed myocardial ischemia-reperfusion injury (Singhanat et al., 2018).

Pyroptosis was first observed in 1992, when Zychlinsky and colleagues noted that macrophages infected with the Gram-negative pathogen *Shigella flexneri* underwent a form of active cell death (Zychlinsky et al., 1992). In 2001, this caspase-1-dependent inflammatory mode of cell death received its formal designation: pyroptosis (Cookson and Brennan, 2001). Pyroptosis is currently defined as gasdermin-mediated programmed cell death (Shi et al., 2017). The human gasdermin family comprises gasdermins A, B, C, and D (GSDMA, GSDMB, GSDMC, and GSDMD), GSDME (DFNA5), and pejvakin (PJVK/DFNB59); notably, mice and rats lack GSDMB (Broz et al., 2020; Dai et al., 2023). With the exception of pejvakin, all gasdermins share a conserved two-domain architecture consisting of an N-terminal domain and a C-terminal domain connected by a flexible linker peptide. Upon activation, caspases or granzymes cleave the gasdermin, liberating the N-terminal domain from the autoinhibitory constraint of the C-terminus. The released N-terminal fragment then oligomerizes at the plasma membrane, forming large pores that permit the release of intracellular contents into the extracellular space. These contents include interleukin-18 (IL-18) and interleukin-1β (IL-1β), which amplify the inflammatory cascade and alert the immune system (Rao et al., 2022; Feng et al., 2018). Beyond the canonical caspase-1 inflammasome pathway, pyroptosis can also be initiated by other caspases that cleave their respective gasdermin substrates. Caspase-3 cleaves GSDME. Caspases-4, -5, -8, and -11 cleave GSDMD. Caspase-6 processes GSDMC (Wang et al., 2017; Orning et al., 2018; Gao et al., 2025; Shamas et al., 2024). Pyroptosis was initially characterized in professional phagocytes, including macrophages, monocytes, and dendritic cells. However, gasdermin-mediated pyroptosis is now recognized to occur in many immune and non-immune cell types, including the cardiomyocytes, cardiac fibroblasts, and vascular endothelial cells discussed below. It can be triggered by intact pathogens, such as bacteria and viruses, or by pathogen-associated molecular patterns, including lipopolysaccharide and viral DNA (Yuan and Ofengeim, 2024). The hallmark of pyroptosis is pore formation in the plasma membrane, irrespective of the upstream trigger. This process culminates in membrane rupture, cell swelling, and the wholesale release of intracellular contents.

## Pathogenesis of myocardial ischemia–reperfusion injury

The molecular mechanisms underlying MIRI remain incompletely defined. When ischemia sets in, cardiomyocytes are abruptly deprived of oxygen, nutrients, and survival factors. As ischemia persists, structural and functional deterioration of the myocardium becomes progressive and ultimately irreversible. The most conspicuous and consequential pathological alteration at this stage is cardiomyocyte death (Kajstura et al., 1996). Reperfusion then subjects the already-compromised tissue to a second wave of insults, including calcium overload, a burst of reactive oxygen species, inflammatory cell infiltration, immune activation, and opening of the mitochondrial permeability transition pore (mPTP) (Turer and Hill, 2010). These pathological events give rise to a spectrum of functional derangements, including reperfusion arrhythmias, myocardial stunning, the no-reflow phenomenon, and lethal reperfusion injury (Figure 1). Lethal reperfusion injury, a form of cell death that manifests only after flow is restored, represents the key driver of infarct expansion (Yellon and Hausenloy, 2007). Emerging evidence has brought pyroptosis to the forefront of MIRI research (Tian et al., 2025).

**FIGURE 1:**
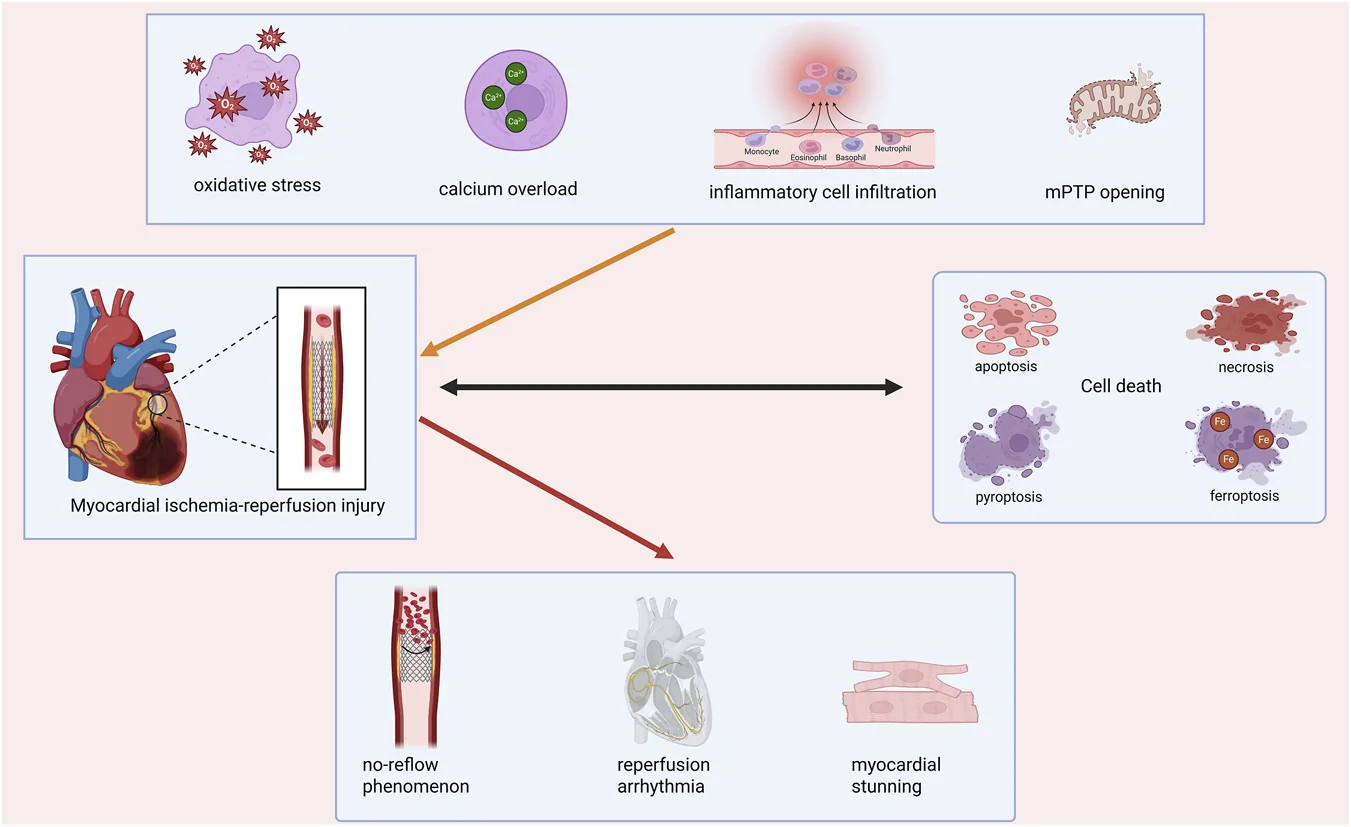
The main pathogenic drivers of MIRI. Created with BioRender.

## Molecular mechanisms of pyroptosis

Pyroptosis is a form of programmed cell death. It shares certain morphological features with apoptosis, including chromatin condensation and DNA fragmentation. However, it diverges sharply in its defining characteristics, which comprise cell swelling, membrane pore formation, osmotic lysis, and the release of pro-inflammatory intracellular contents (Kroemer et al., 2009; Kepp et al., 2010; Chen et al., 2016). GSDMD serves as the core substrate for inflammatory caspases, including caspase-1, 4, 5, 11. These caspases cleave GSDMD at the linker region connecting its N- and C-terminal domains, thereby dismantling the molecule’s autoinhibitory conformation and liberating the pyroptosis-inducing N-terminal fragment. In the absence of GSDMD, neither cytosolic lipopolysaccharide (LPS) nor inflammasome ligands can trigger pyroptosis (Shi et al., 2015). GSDMD therefore stands as the central executioner of pyroptosis. The pathways that converge upon its activation fall into two arms. The canonical route is driven by NLRP3 and other classical inflammasomes. The non-canonical route provides an alternative activation mechanism (Figure 2).

**FIGURE 2:**
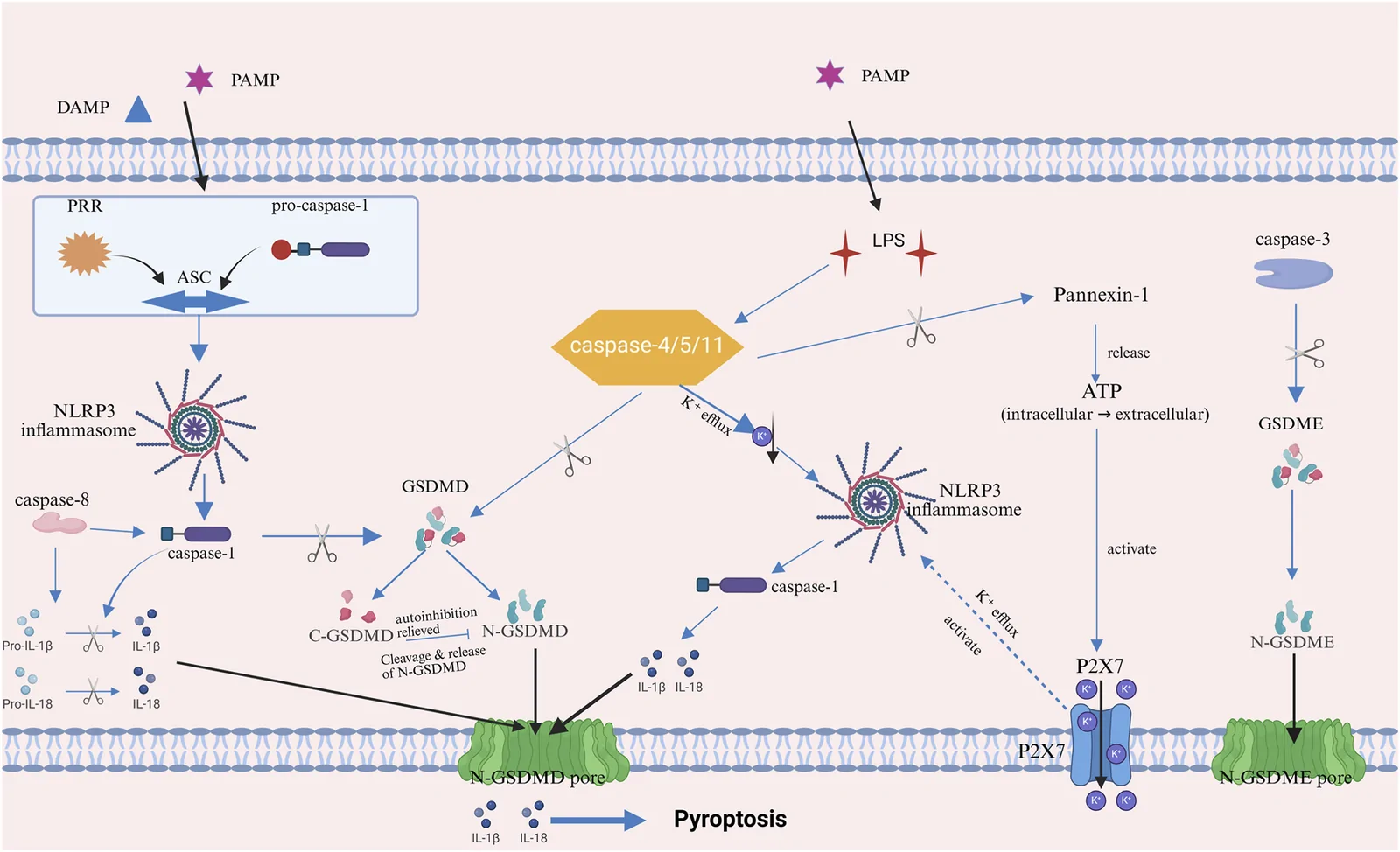
Common signaling pathways of pyroptosis. Abbreviations: DAMP, damage-associated molecular pattern; PAMP, pathogen-associated molecular pattern; PRR, pattern recognition receptor; ASC, apoptosis-associated speck-like protein containing a CARD; GSDMD, gasdermin D; C-GSDMD, C-terminal fragment of gasdermin D; N-GSDMD, N-terminal fragment of gasdermin D; GSDME, gasdermin E; N-GSDME, N-terminal fragment of gasdermin E; IL-1β, interleukin-1β; IL-18, interleukin-18; LPS, lipopolysaccharide. Created with BioRender.

### The canonical pathway

The canonical pathway depends on inflammasome assembly, which primes the machinery for GSDMD cleavage and the subsequent release of IL-18 and IL-1β (Vasudevan et al., 2023; Fang et al., 2020; Panganiban et al., 2024; Xiao et al., 2024). Inflammasomes are multiprotein signaling platforms that assemble upon sensing microbial pathogens or danger signals, placing them at the center of innate immune regulation (Broz and Dixit, 2016). Their significance extends beyond infectious disease, for example, inflammasomes and their downstream mediators are now implicated in cancer, hematologic disorders, and MIRI (Deng et al., 2023; Tomasik and Basak, 2022; Algoet et al., 2023). Inflammasome assembly is initiated by cytosolic pattern recognition receptors (PRRs) that detect pathogen-associated molecular patterns (PAMPs) and damage-associated molecular patterns (DAMPs) (Paerewijck and Lamkanfi, 2022). Upon detection of bacterial or viral signals, PRRs bind pro-caspase-1 and apoptosis-associated speck-like protein containing a CARD (ASC), subsequently assembling into inflammasomes (Vasudevan et al., 2023; Christgen and Kanneganti, 2020).

The best-characterized inflammasomes include NLRP1, NLRP3, NLRC4, AIM2, and pyrin, all of which are dependent on caspase-1, as well as the caspase-11 non-canonical inflammasome. A typical inflammasome is assembled from three components: (i) a leucine-rich repeat-containing sensor protein (NLR), (ii) the adaptor ASC, and (iii) pro-caspase-1 (Triantafilou, 2021). NLR proteins typically contain a leucine-rich repeat (LRR), a nucleotide-binding oligomerization domain (NACHT), and either a caspase activation and recruitment domain (CARD) or a pyrin domain (PYD). Based on their terminal domain, NLRs fall into two subfamilies: NLRCs, which carry one or more CARD domains at the N-terminus, and NLRPs, which carry a PYD domain (Yu et al., 2021). The adaptor ASC contains both a PYD domain and a CARD domain. Activated sensor proteins that bear a PYD domain recruit ASC via homotypic PYD–PYD interactions; ASC, in turn, recruits pro-caspase-1 through CARD–CARD interactions (Vasudevan et al., 2023).

Among these, the NLR-family inflammasomes have been studied most extensively; however, both AIM2 and pyrin are also capable of nucleating inflammasome assembly. Once assembled, the inflammasome recruits and activates caspase-1 via autocatalytic cleavage (Okin and Kagan, 2023). Activated caspase-1 then cleaves GSDMD, forming pores in the plasma membrane through which IL-18 and IL-1β are released. Concurrently, caspase-1 processes pro-IL-18 and pro-IL-1β into their mature forms, thereby fueling the inflammatory cascade that culminates in pyroptosis (Liu Y. et al., 2024; Zhang W-J. et al., 2021).

### The non-canonical pathway

In the non-canonical pyroptosis pathway, human caspase-4 and caspase-5 (or murine caspase-11) directly bind lipopolysaccharide (LPS) via their N-terminal CARD domains, thereby triggering activation (Kovacs and Miao, 2017). Activated caspase-4, caspase-5, and caspase-11 then cleave GSDMD, separating the N-terminal domain from the C-terminal domain to relieve the autoinhibitory constraint imposed by the C-terminus. The liberated N-terminal fragment subsequently oligomerizes in the plasma membrane, forming pores that induce cell swelling and membrane lysis, which in turn mediate the release of cellular contents and exacerbate inflammation (Zheng et al., 2021a). Notably, caspase-4, caspase-5, and caspase-11 cannot directly cleave pro-IL-18 or pro-IL-1β; instead, they engage the classical NLRP3/caspase-1 pathway to promote the maturation and secretion of these cytokines (Li et al., 2023). Mechanistically, LPS-activated caspase-11 cleaves GSDMD, and the resulting potassium efflux accelerates NLRP3 inflammasome assembly, ultimately triggering pyroptosis (Rühl and Broz, 2015). Pannexin-1 represents another key mediator of non-canonical caspase-11-induced pyroptosis. Upon cleavage by caspase-11, Pannexin-1 mediates the release of ATP, which in turn activates the P2X7 receptor, driving further potassium efflux, inflammasome formation, and the induction of pyroptosis (Yang et al., 2015).

Current evidence indicates that caspase-3 can specifically cleave and release the N-terminal domain of GSDME, which binds to plasma membrane lipids such as PIP2 and cardiolipin to form pores, thereby eliciting the characteristic morphological features of pyroptosis, including cell vesiculation, swelling, and lysis (Wang et al., 2017). The chimeric antigen receptor T (CAR-T) cells, which can rapidly activate caspase-3 in target cells by releasing granzyme B. This subsequently triggers the caspase-3/GSDME-mediated pyroptotic pathway, leading to massive cell pyroptosis. The damage signals released during pyroptosis then further activate the canonical caspase-1/GSDMD pyroptotic pathway in macrophages (Liu et al., 2020). The mode of cell death shifts depending on the level of GSDME expression. When GSDME is expressed at low levels, cells predominantly undergo apoptosis, whereas high GSDME expression converts apoptosis into pyroptosis.

Caspase-8 activation promotes caspase-1 activation, which in turn triggers GSDMD cleavage and pyroptosis. Concurrently, it can block the RIPK3/MLKL necroptotic pathway, thereby diverting cell death toward pyroptosis (Cui et al., 2024). In models of TNF-induced systemic inflammatory response syndrome (SIRS) and sepsis, caspase-8 has been shown to directly mediate IL-1β maturation independently of the NLRP3/caspase-1 pathway (Muendlein et al., 2025). Current evidence indicates that caspase-8 functions as a molecular switch governing apoptosis, necroptosis, and pyroptosis. When its enzymatic activity is lost, caspase-8 can recruit ASC and activate caspase-1 through its scaffolding function, thereby engaging the classical inflammasome pathway (Fritsch et al., 2019). TRPM7 mediates GSDMD-dependent pyroptosis by regulating Zn^2+^ homeostasis, activating the ROS-JAK2/STAT3 pathway, upregulating NLRP3, and initiating the classical inflammasome pathway (Tong et al., 2024).

In 2020, Zhou et al. reported that cytotoxic lymphocytes, including natural killer cells and cytotoxic T lymphocytes (NK/CTL), can secrete granzyme A (GZMA), which directly cleaves GSDMB and induces pyroptosis in target cells. This discovery overturned the prevailing notion that pyroptosis can only be triggered by caspase activation (Zhou Z. et al., 2020).

## Cellular mediators of pyroptosis in myocardial ischemia-reperfusion injury

The heart is a multicellular organ composed of 11 major cell types, including cardiomyocytes, cardiac fibroblasts, vascular endothelial cells, and resident immune cells (Litviňuková et al., 2020). Among these, cardiomyocytes, cardiac fibroblasts, and vascular endothelial cells constitute the majority of cells in the myocardial tissue (Litviňuková et al., 2020). After myocardial infarction, increased caspase-1 activity and aggregation of inflammasome components in the cytoplasm provide direct evidence of inflammasome assembly in the damaged myocardium (Mezzaroma et al., 2011). NLRP3 expression is upregulated in cardiac fibroblasts, which is involved in mediating myocardial ischemia–reperfusion injury (Sandanger et al., 2013). In the setting of MIRI, cell death does not occur synchronously or to the same degree across all cardiac cell types. The cell types differ in their major triggers, the signaling pathways activated, and the functional outcomes of cell death. Cardiomyocyte pyroptosis leads to the loss of contractile cells, directly precipitating pump failure; fibroblast pyroptosis amplifies the fibrotic response; endothelial cell pyroptosis disrupts the microcirculation and promotes the no-reflow phenomenon; and macrophage pyroptosis further drives the inflammatory cascade, exacerbating the various types of injury described above. Accordingly, the following sections will address each cell type separately. However, the existing evidence is not evenly distributed across cell types. As noted above, most studies have focused on GSDMD, whereas data on other gasdermin family members in different cell types remain scarce—an important limitation that persists in this field.

### Cardiomyocytes

Cardiomyocytes are terminally differentiated cells with negligible regenerative capacity, and their death is closely linked to MIRI. During MIRI, cardiomyocytes can undergo pyroptosis through both the caspase-1/GSDMD and caspase-11/GSDMD pathways. Current evidence further demonstrates that GSDMD plays a key role in cardiomyocyte pyroptosis. In mice, GSDMD and caspase-11 were significantly elevated at both 0.5 h and 24 h after reperfusion, and were predominantly accumulated in cardiomyocytes. Knockout of the GSDMD gene has been shown to significantly reduce myocardial infarct size while concurrently inhibiting MIRI-induced cardiomyocyte pyroptosis and the release of IL-18. Under conditions of oxidative stress, pyroptosis in cardiomyocytes is primarily regulated by the caspase-11/GSDMD signaling pathway (Shi et al., 2021; Wang et al., 2025). Moreover, in an *in vivo* MIRI mouse model and in primary cardiomyocytes subjected to H/R *in vitro*, consistent and significant upregulation of NLRP3, ASC, caspase-1, GSDMD, and IL-18 has been observed in the early phase of ischemia and reperfusion; downregulation of these pyroptosis-related factors significantly reduces myocardial infarct size and lowers circulating cardiac injury markers (Liu S. et al., 2024; Zhang D. et al., 2024). In a mouse model of AMI, cardiomyocyte-specific BMP7 expression blocks NF-κB pathway activation by inhibiting IκBα degradation and preventing p65 nuclear translocation, thereby downregulating the expression and assembly of the NLRP3 inflammasome and suppressing GSDMD-mediated pyroptosis in cardiomyocytes, in other words, it modulates the NF-κB/NLRP3/GSDMD signaling axis. BMP7 can also inhibit cardiomyocyte pyroptosis, thereby reducing the upstream injury signals that drive fibroblast activation at their source, including paracrine damage signals such as IL-1β and IL-18. As a result, the transdifferentiation of fibroblasts into myofibroblasts is suppressed, and pathological fibrosis is alleviated. Clinical sample analyses have revealed that circulating BMP7 levels are significantly reduced in the peripheral plasma of patients with chronic MI (Liu et al., 2026). Additionally, cardiomyocyte-specific TRPM7 intracellular vesicles respond to oxidative stress by mediating Zn^2+^ release, which in turn triggers pyroptosis and ultimately promotes cardiomyocyte death during the process of ischemia–reperfusion injury (Li et al., 2026). In addition to the GSDMD pathway, we also found that aerobic exercise inhibits cardiomyocyte pyroptosis by suppressing the GSDME/caspase-3 pathway after reperfusion (24 h). Moreover, GSDME knockout mice showed improved left ventricular function and reduced infarct size (Li Y. et al., 2024). However, in the specific setting of MIRI, direct functional validation of GSDME remains largely limited to cardiomyocytes, and its precise roles in cardiac fibroblasts and endothelial cells have not yet been fully delineated. Cardiomyocyte pyroptosis is involved in both the ischemic and reperfusion phases of MIRI. Moreover, pyroptosis of other cell types can also affect cardiomyocytes. Ultimately, this reduces the number of functional cardiomyocytes and impairs cardiac contractile function. However, many existing studies have limitations. They have not dynamically observed the changes in NLRP3 and its downstream effectors in cardiomyocytes during reperfusion, and most of the research has been conducted in cells and small animals. Future studies using large animal models may therefore be needed to provide new value for clinical practice.

### Cardiac fibroblasts

Cardiac fibroblasts are the most abundant non-cardiomyocyte cell type in cardiac tissue and play a crucial role in maintaining cardiac function. Upon tissue injury or inflammatory stimulation, cardiac fibroblasts become activated, thereby contributing to the regulation of the inflammatory response and the mediation of tissue repair (Cadosch et al., 2024).

Cardiac fibroblast pyroptosis impairs cardiac function primarily by amplifying the inflammatory response and promoting myocardial fibrosis. In cardiac fibroblasts, the levels of NLRP3, caspase-1, cleaved caspase-1, GSDMD-N, IL-1β and a panel of inflammatory cytokines (IL-6, IL-18, TNF-α) rise concurrently and progressively with prolonged reperfusion time, peaking at 24 h post-reperfusion. Furthermore, inhibition of the NLRP3 signaling pathway attenuates pyroptosis in cardiac fibroblasts, suppresses inflammatory cytokine secretion and cardiac inflammatory response, and reduces myocardial infarct size (Zhang M. et al., 2021). During reperfusion, cardiac fibroblasts can further activate the inflammasome via reactive oxygen species (ROS) generation and K^+^ efflux, which in turn drives IL-1β release, recruits inflammatory cells, and exacerbates inflammatory injury (Kawaguchi et al., 2011). Recent studies have established that epigenetic mechanisms contribute to the progression of cardiac fibrosis by regulating pyroptosis in cardiac fibroblasts. Specifically, DNA methyltransferase 3 A (DNMT3A)-mediated DNA methylation silences the lncRNA Neat1. This event derepresses the NLRP3 inflammasome signaling pathway, drives pyroptosis in cardiac fibroblasts, and consequently promotes the progression of cardiac fibrosis (Ding et al., 2023). In addition, the long non-coding RNA GAS5 activates the NLRP3 inflammasome by silencing DNMT1, thereby similarly promoting fibroblast pyroptosis and exacerbating cardiac fibrosis (She et al., 2020). ALKBH5 (an m^6^A RNA demethylase) promotes NLRP3 activation by regulating the m^6^A modification of Notch1 (an NLRP3-associated molecule), and thereby modulates pyroptosis in cardiac fibroblasts. Following MI, cardiac fibroblasts shuttle damaged mitochondrial components out of the cell via small extracellular vesicles (sEVs) — referred to as CFs-mt-sEVs. Upon uptake by macrophages, these sEVs promote macrophage polarization toward a pro-inflammatory phenotype through NLRP3 activation, thereby exacerbating tissue inflammation, cardiac dysfunction, and adverse ventricular remodeling (Zhao et al., 2025). Pyroptotic cardiomyocytes, macrophages and other cell types secrete abundant IL-1β as a key paracrine signal that targets cardiac fibroblasts, promoting fibroblast proliferation, collagen synthesis, matrix metalloproteinase (MMP) expression and other downstream cellular responses, thereby driving myocardial fibrosis (Turner, 2014). However, most existing studies are predominantly based on *in vitro* cellular experiments and animal gene knockout models, with limited translational evidence for clinical application.

### Vascular endothelial cells

The cardiac microvascular endothelium is a critical component of the myocardial microcirculation, and its role in counteracting MIRI and protecting cardiomyocytes has garnered increasing attention. Endothelial cells undergo pyroptosis via multiple distinct pathways, the most well-characterized of which is the canonical NLRP3 axis. In human umbilical vein endothelial cells (HUVECs) subjected to H/R treatment, the expression levels of IL-1β, NLRP3 inflammasome components, cleaved (activated) caspase-1, and GSDMD are all elevated (Wu et al., 2025). Cardiac microvascular endothelial cells (CMECs) also undergo pyroptosis via this signaling axis, with marked impairment of proliferation, migration, angiogenic capacity and secretory function, which further exacerbates cardiac dysfunction and adverse myocardial remodeling (Zhang et al., 2022). Upstream of this signaling axis, endothelial cell-specific S1PR2 induces aberrant mitochondrial fragmentation in endothelial cells via the RHO/ROCK1/DRP1 signaling pathway, which in turn activates NLRP3 and triggers endothelial pyroptosis (Duan et al., 2024).

Following I/R, the expression level of high mobility group box 1 (HMGB1) rises progressively with prolonged reperfusion time. In the early phase (within 24 h post-reperfusion), the HMGB1 response is primarily driven by endothelial cells and cardiomyocytes, whereas in the late phase (3 days post-reperfusion onwards), it is dominated by fibroblasts. HMGB1 induces pyroptosis in endothelial cells (ECs) via activation of the AIM2 inflammasome, which disrupts microcirculatory homeostasis and exacerbates inflammation and oxidative stress, thereby aggravating myocardial injury. Meanwhile, EC-derived HMGB1 may also participate in regulating HMGB1 protein levels in post-ischemic cardiomyocytes and fibroblasts through paracrine signaling or intercellular signal transduction mechanisms (Chen R. et al., 2025). HMGB1 is a profibrotic paracrine mediator released upon cellular pyroptosis. Inhibition of HMGB1 ameliorates myocardial fibrosis, and this effect is partially mediated via endothelial-to-mesenchymal transition (EndMT) (Zhang et al., 2019). HMGB1 is also increasingly recognized as a therapeutic target for myocardial fibrosis (Dai et al., 2021). In a clinical study, serum HMGB1 levels peaked at 24 h post-reperfusion in patients with STEMI undergoing PCI (Sørensen et al., 2011). Further in-depth clinical studies may validate its utility as an additional biomarker for myocardial injury. Both I/R and H/R not only induce the expression of fatty acid-binding protein 3 (FABP3) in CMECs, thereby activating the canonical pyroptosis pathway, but also trigger mitochondrial damage and mitochondrial DNA (mtDNA) release via the YTHDF1/E2F8/FABP3 signaling axis, which in turn activates cGAS-STING signaling. This pathway can both directly trigger pyroptosis and amplify the pyroptotic effect through inflammatory cell infiltration and ROS generation (Sha et al., 2026). Furthermore, the non-canonical pyroptosis pathway also functions in CMECs, I/R activates caspase-4 in these cells independently of inflammasome assembly, which in turn drives IL-1β release and GSDMD upregulation (Sun et al., 2021). Following ECs pyroptosis, neutrophils further exacerbate endothelial injury. In MIRI, the inflammatory response upregulates adhesion molecule expression, thereby promoting neutrophil adhesion and infiltration. Free radicals and lysosomal enzymes released by neutrophils disrupt endothelial integrity, leading to microthrombosis, tissue edema, and the no-reflow phenomenon (Hou et al., 2022; Korayem et al., 2017). Taken together, injured ECs undergo pyroptosis via multiple distinct pathways, which ultimately exacerbates myocardial injury. However, most relevant studies are largely restricted to murine and cellular models, which oversimplify the complex pathophysiological processes in clinical settings. Further in-depth clinical investigations in this area are warranted in the future. Furthermore, mitochondrial damage emerges repeatedly as an upstream event across multiple pathways, suggesting that it may be a prerequisite for multiple endothelial pyroptosis pathways and could serve as a potential interventional target in future investigations.

### Macrophages

Macrophage dysfunction is a primary driver of the excessive inflammatory response in MIRI and a key contributor to the pathogenesis of microvascular reperfusion injury. After MIRI, macrophages display increased levels of cleaved GSDMD and extensive pyroptosis, which promote the secretion of inflammatory cytokines, accelerate neutrophil infiltration, and elevate ROS production, thereby exacerbating oxidative stress (Ye et al., 2022). Macrophages can promote myocardial injury through NLRP3/caspase-1 mediated pyroptosis. For instance, stachyose inhibits macrophage pyroptosis by blocking this pathway, thereby reducing the production of inflammatory cytokines such as IL-18 and IL-1β, which in turn limits infarct size and improves cardiac function (Zhang A-Y. et al., 2024). NLRP3 inflammasome activation and pyroptosis in macrophages triggered by pro-inflammatory stimuli not only amplify the inflammatory response but also further aggravate damage to CMECs (Sun et al., 2023). Following MIRI, macrophages are extensively recruited and activated, leading to aberrantly high expression of NOX1 and NOX4. This shift drives mitochondria to produce abundant ROS, precipitates mitochondrial dysfunction, and suppresses mitochondrial energy metabolism. These events, in turn, activate the NLRP3 inflammasome, inducing pyroptosis in cardiomyocytes and immune cells, particularly macrophages. Pyroptotic cells then release large quantities of pro-inflammatory factors, including IL-1β and IL-18, which amplify the local myocardial inflammatory response and further exacerbate myocardial injury (Pan et al., 2025). Macrophages serve as a key cellular nexus bridging acute inflammation and fibrotic remodeling following MIRI. During the acute phase (2 h post-reperfusion), they amplify the inflammatory cascade via the MyD88/NF-κB/NLRP3 signaling axis, whereas during the reparative phase at 3–7 days post-reperfusion, they promote the transition of both macrophages themselves and fibroblasts into myofibroblasts, thereby driving and exacerbating myocardial fibrosis (Shen et al., 2024). Cardiac macrophages comprise at least two distinct cell populations of entirely different origins: tissue-resident macrophages that populate the heart prior to injury (Litviňuková et al., 2020), and monocyte-derived macrophages recruited to the heart from the peripheral circulation following the onset of ischemia (Pan et al., 2025). The relative proportions of these two populations shift dynamically over the course of the injury response. Within this dynamically changing cell pool, individual macrophages are not fixed in either an M1 or M2 phenotype; instead, their functional state remodels in response to the local microenvironment (Murray et al., 2014). Activated IL-1β binds to the IL-1 receptor (IL-1R) on cardiac resident macrophages, which augments caspase-1 maturation and in turn drives increased production of IL-1β, forming a self-amplifying positive feedback loop (Moreno-Loaiza et al., 2025); however, this mechanism was demonstrated in an atrial fibrillation model, and whether the same loop operates in resident cardiac macrophages during MIRI remains to be confirmed. MIRI also triggers the release of large numbers of I/R-induced cardiac extracellular vesicles (EVs), which not only promote local sterile inflammation in the heart, but can also induce systemic inflammation. For example, EVs released after MIRI can deliver enriched miR-155–5p to macrophages, where it activates the JAK2/STAT1 signaling pathway and drives a pro-inflammatory phenotype in macrophages. This promotes both local cardiac and systemic inflammation, ultimately aggravating myocardial injury (Ge et al., 2021). Conversely, exosomes derived from M2-like macrophages polarized by IL-4 induction (M2-exos) can transfer miR-148a into cardiomyocytes, where it directly targets and downregulates TXNIP. This in turn suppresses the TLR4/NF-κB/NLRP3 inflammasome signaling pathway, thereby inhibiting cardiomyocyte pyroptosis, reducing the release of inflammatory cytokines, and alleviating Ca^2+^ overload, ultimately attenuating MIRI-induced myocardial injury (Dai et al., 2020). Stem-cell transplantation represents a vital strategy for cardiac regeneration after MI. In hearts subjected to I/R injury, M1-macrophages recruited to the injured site secrete IL-1β, which induces pyroptosis in transplanted stem cells via paracrine signaling. This process constitutes one of the major causes of acute stem-cell death following transplantation. MiR-762 exerts a protective effect by specifically inhibiting IL-1β and consequently blocking pyroptosis (Lee et al., 2021). In sepsis, macrophage-derived exosomes carrying miR-141 can target and activate NLRP3, thereby inducing pyroptosis of endothelial cells (Zhan et al., 2024). Whether this exosome-mediated pathway also exists in MIRI has not yet been verified; nevertheless, the shared dependence on NLRP3 in both conditions suggests that this pathway may well be operative in MIRI. Macrophage pyroptosis not only aggravates inflammation by directly promoting pyroptosis, but also exacerbates MIRI indirectly by inducing damage to CMECs and worsening mitochondrial dysfunction, thereby enlarging the infarct size and impairing cardiac function. Nevertheless, whether this pathway promotes endothelial cell pyroptosis in MIRI requires further confirmation. In addition, macrophages and cardiac fibroblasts can interact with each other after MIRI, further amplifying the inflammatory response and fibrosis.

### Pyroptotic crosstalk across cardiac cell populations in MIRI

Pyroptosis in MIRI is not a purely cell-autonomous event. Because gasdermin-mediated lysis releases a broad repertoire of intracellular contents (Rao et al., 2022; Feng et al., 2018), pyroptosis occurring in one cardiac cell population can reprogram the survival, phenotype, and secretory activity of neighboring populations, thereby converting a focal cell-death event into a self-propagating, tissue-wide inflammatory response. Three classes of messengers dominate this crosstalk: the cytokines IL-1β and IL-18; damage-associated molecular patterns (DAMPs) such as HMGB1, ATP, and mitochondrial DNA; and EVs, which transfer microRNA and organelle cargo between cardiac cell populations (Zhao et al., 2025; Ge et al., 2021). Pyroptotic cells are not merely passive sources of these signals; rather, EVs shed by pyroptotic cells can transplant pre-assembled GSDMD pores onto the plasma membrane of bystander cells, propagating lytic death in a domino-like manner (Wright et al., 2025), thus establishing EVs as *bona fide* vectors of pyroptotic spread. Temporally, these pathways organize pyroptotic signaling into a structured relay—an acute pyroptotic wave (0.5–24 h) in cardiomyocytes, endothelial cells, and macrophages (Shi et al., 2021; Wu et al., 2025; Ye et al., 2022); a fibrotic switch at approximately 24 h, when pyroptotic markers in cardiac fibroblasts reach their peak (Zhang M. et al., 2021); and a reparative phase (3–7 days), during which macrophages drive the transition of fibroblasts and macrophages into myofibroblasts (Shen et al., 2024).

Recruited macrophages serve as the principal upstream amplifiers of this relay. M1 macrophage-derived IL-1β acts as a paracrine death signal that directly induces pyroptosis in cardiomyocytes. However, microRNA miR-762 interrupts this route by inhibiting IL-1β (Lee et al., 2021). Under H/R conditions, M1 macrophage-derived exosomes reinforce this route. They deliver miR-29a, which downregulates MCL-1 and induces cardiomyocyte pyroptosis (Wang et al., 2021). Macrophage-derived reactive oxygen species also act on the same axis. After MIRI, aberrantly elevated NOX1/NOX4 expression drives mitochondrial ROS production and NLRP3 activation. This in turn induces pyroptosis in both cardiomyocytes and immune cells themselves (Pan et al., 2025). NLRP3 activation and pyroptosis in macrophages directly exacerbate injury to cardiac microvascular endothelial cells (Sun et al., 2023). This indirectly compromises cardiomyocyte perfusion. Within the macrophage pool itself, IL-1β binds to the IL-1 receptor on resident macrophages. This binding augments caspase-1 maturation and further IL-1β production, forming a self-amplifying positive feedback loop (Moreno-Loaiza et al., 2025). This relay can also be ignited remotely. Notably, this regulatory circuit was demonstrated in an atrial fibrillation model, and whether this same loop functions during MIRI remains to be confirmed. ATP is released into the circulation from a reperfused ischemic liver. It then activates P2X7 receptors on cardiac macrophages. These receptors in turn drive GSDMD-dependent cardiomyocyte pyroptosis through IL-1β (Zhang et al., 2026). Together, these findings indicate that macrophage-amplified pyroptotic signaling can propagate beyond the ischemic heart itself.

Cardiac fibroblasts are principal recipients of pyroptotic paracrine signals. IL-1β is secreted by pyroptotic cardiomyocytes and macrophages. This cytokine drives fibroblast proliferation, collagen synthesis, and matrix metalloproteinase expression (Turner, 2014). Conversely, cardiomyocyte-derived BMP7 suppresses pyroptosis-derived paracrine IL-1β/IL-18. Through this action, BMP7 inhibits fibroblast-to-myofibroblast transdifferentiation and pathological fibrosis (Liu et al., 2026). Endothelial injury feeds the same fibrotic program through the DAMP axis. During the first 24 h of reperfusion, endothelial cells and cardiomyocytes are the dominant sources of myocardial HMGB1. Endothelial-derived HMGB1 regulates HMGB1 in post-ischemic cardiomyocytes and fibroblasts via paracrine signaling (Chen R. et al., 2025). HMGB1 released upon pyroptosis acts as a pro-fibrotic mediator. Inhibition of HMGB1 ameliorates fibrosis, in part by suppressing endothelial-to-mesenchymal transition (EndMT) (Zhang et al., 2019; Dai et al., 2021). From 3 days onward, the dominant source of HMGB1 shifts to fibroblasts themselves (Chen R. et al., 2025). This indicates that pyroptotic fibroblasts sustain the pro-fibrotic milieu during later remodeling.

Extracellular vesicles (EVs) complete this network as a dedicated route of signal transfer. I/R-derived cardiac EVs deliver miR-155–5p to macrophages. This drives a pro-inflammatory phenotype and propagates local and systemic inflammation (Ge et al., 2021). Fibroblast-derived small EVs carry damaged mitochondria (CFs-mt-sEVs). These EVs activate the NLRP3 inflammasome in macrophages and skew them toward a pro-inflammatory phenotype (Zhao et al., 2025). As a protective counter-arm, M2-derived exosomes deliver miR-148a into cardiomyocytes. This restrains the TLR4/NF-κB/NLRP3 axis (Dai et al., 2020). In addition, M2-derived exosomal miR-378a-3p suppresses post-infarction cardiomyocyte pyroptosis. It does so by destabilizing NLRP3 through inhibition of HuR (Yuan et al., 2022). Cardiac fibroblasts are not only recipients but also senders of pyroptotic signals. During hypoxia/reoxygenation, cardiac fibroblasts secrete exosomes that deliver miR-133a into cardiomyocytes. There, miR-133a targets ELAVL1, which suppresses pyroptosis and alleviates myocardial ischemia/reperfusion injury (Liu N. et al., 2022). In sepsis, macrophage-derived exosomes carrying miR-141 can induce endothelial pyroptosis. However, this route remains to be verified in MIRI (Zhan et al., 2024). Taken together with the EV-borne transfer of GSDMD pores described above (Wright et al., 2025), these vesicular routes define a bidirectional transport system. This system is pro-inflammatory from the parenchyma to macrophages. It is protective from M2 macrophages back to the parenchyma. It is also supplemented by protective signals from cardiac fibroblasts and endothelial cells to cardiomyocytes. This network could therefore be exploited therapeutically at the level of EV cargo or uptake.

Notably, this network contains both positive-feedback loops and endogenous protective arms. The positive-feedback loops include macrophage IL-1β self-amplification (demonstrated in an atrial fibrillation model) (Moreno-Loaiza et al., 2025) and neutrophil–endothelium mutual injury, which sustains ischemia through the resulting no-reflow (Hou et al., 2022; Korayem et al., 2017). The protective arms include BMP7, miR-762, and M2-derived miR-148a (Liu et al., 2026; Lee et al., 2021; Dai et al., 2020). These observations suggest that interrupting pyroptotic propagation at intercellular checkpoints—such as IL-1β/IL-1 receptor signaling, HMGB1, or EV-mediated cargo transfer—may complement the cell-intrinsic inhibitors discussed below. However, the receptor repertoires and *in vivo* kinetics of these routes remain largely undefined. Delineating them will require cell-type-specific genetic tools, single-cell and spatial transcriptomic mapping, and dynamic EV tracking.

## Mechanisms and regulation of pyroptosis in myocardial ischemia-reperfusion injury

### Oxidative stress

Oxidative stress refers to an imbalance between the body’s oxidative and antioxidant systems, favoring a pro-oxidative state that leads to the excessive accumulation of ROS and subsequent cellular and tissue damage. During myocardial ischemia and reperfusion, large quantities of ROS are generated. The accumulated ROS activate the canonical NLRP3 axis. This process mediates pyroptosis and exacerbates myocardial tissue damage and the no-reflow phenomenon (Nie et al., 2021a; Qiu et al., 2019). Excessive ROS accumulation activates Drp1 to trigger mitochondrial fission, while simultaneously stimulating NOX2 to generate additional ROS. Drp1 and NOX2 thus become locked in a vicious cycle that sustains NLRP3 inflammasome activation and the release of IL-1β and IL-18 (Li et al., 2016). Furthermore, following ROS-induced pyroptosis, NLRP3 inflammasome activation may directly promote further ROS production or be indirectly amplified by other NLRP3 downstream factors such as IL-18, thereby perpetuating this vicious cycle (Liu Y. et al., 2022). Wang et al. demonstrated that trehalose significantly suppresses ROS generation in cardiomyocytes, thereby blocking the canonical NLRP3 axis mediated pyroptosis. This attenuation of pyroptosis improves cardiac dysfunction and reduces infarct size in mice (Wang et al., 2024). Collectively, these findings indicate that oxidative stress in MIRI triggers pyroptosis primarily through the NLRP3/caspase-1/GSDMD pathway, generating abundant inflammatory mediators that amplify the inflammatory response and exacerbate myocardial injury. Studies have shown that Nuclear Factor Erythroid 2-Related Factor 2 (Nrf2) reduces ROS production. Sweroside binds to and inhibits KEAP1, thereby promoting NRF2 nuclear translocation. NRF2 subsequently activates antioxidant genes, reducing ROS levels and alleviating oxidative stress. This cascade inhibits NLRP3 inflammasome activation, attenuates pyroptosis, and ultimately ameliorates cardiac injury while improving cardiac function (Li et al., 2021). In both *in vitro* and *in vivo* models of MIRI, miR-132 is upregulated. It specifically inhibits Silent Information Regulator 1 (SIRT1), thereby suppressing the PGC-1α/Nrf2 antioxidant pathway. This suppression enhances oxidative stress and activates the NLRP3 pyroptosis pathway, ultimately exacerbating myocardial injury. Notably, inhibition of miR-132 can reverse these deleterious effects (Zhou Y. et al., 2020).

### Calcium overload

During MIRI, factors such as ATP depletion and disruption of ion transport systems lead to the substantial accumulation of Ca^2+^ within the cytosol and mitochondria, resulting in calcium overload. Excessive intracellular calcium can precipitate mitochondrial dysfunction and metabolic disturbances, promote the production of ROS, and induce arrhythmias, thereby exacerbating myocardial damage (Zhang S. et al., 2024). MIRI also triggers the massive accumulation of ROS and Ca^2+^, which synergistically promote the opening of the mPTP. Once opened, the mPTP not only exacerbates ROS production through a positive feedback mechanism but also facilitates the oxidation of mitochondrial DNA (mtDNA) to generate oxidized mtDNA (ox-mtDNA), which is subsequently released into the cytoplasm. In turn, ox-mtDNA activates caspases- GSDMs axis, ultimately inducing multiple forms of cell death including pyroptosis. (Mas et al., 2025). The mitochondrial permeability transition pore (mPTP) is regarded as the natural convergence point of these signals; however, the clinical translation of this theory has met with failure. For example, cyclosporin A is a potent mPTP inhibitor in preclinical studies; however, the CIRCUS trial showed that administration of cyclosporin A prior to PCI did not reduce infarct size (Cung et al., 2015). Emerging evidence indicates that calcium overload is closely linked to cardiomyocyte pyroptosis. Such as Inositol 1,4,5-trisphosphate receptor (IP3R), an intracellular calcium release channel, is upregulated in the MIRI model. Elevated IP3R expression induces intracellular calcium overload, which subsequently promotes pyroptosis through activation of the canonical NLRP3 axis. Conversely, artificial upregulation of endoplasmic reticulum resident protein 44 (ERP44) or administration of the calcium channel blocker nifedipine D6 to attenuate calcium flux markedly downregulates pyroptosis-associated markers, including NLRP3 and caspase-1 (Mo et al., 2021). Zheng et al. reported that dexmedetomidine (DEX) specifically inhibits CaMKIIδ by upregulating intracellular miR-346–3p, thereby alleviating calcium overload, suppressing the NF-κB/NLRP3 pathway, and ultimately attenuating cardiomyocyte pyroptosis and reducing infarct size (Zheng et al., 2021b). In addition to the classical NLRP3 signaling pathway, calcium overload can also promote pyroptosis via the caspase-3/GSDME pathway. Zhou et al. demonstrated glioblastoma cells that lidocaine induces phosphorylation of transient receptor potential vanilloid 1 (TRPV1), which in turn provokes calcium overload, a decline in mitochondrial membrane potential, and increased caspase-3 release. Caspase-3 subsequently cleaves GSDME, culminating in pyroptosis (Zhou et al., 2021); however, this TRPV1/caspase-3/GSDME axis was established in a non-cardiac model, and whether it operates in cardiomyocytes during MIRI remains to be confirmed. Calcium overload not only induces pyroptosis but also promotes ROS production, thereby triggering oxidative stress; in the context of MIRI, oxidative stress and pyroptosis engage in a positive feedback loop, reciprocally exacerbating injury. Collectively, these findings highlight the close interrelationship among calcium overload, oxidative stress, and pyroptosis in the pathogenesis of MIRI.

### Autophagy

Autophagy inhibits pyroptosis and alleviates MIRI. Autophagy and pyroptosis are mutually antagonistic in MIRI; however, their interplay is dose-dependent. Under physiological conditions, it can suppress inflammasome formation through multiple mechanisms. Specifically, autophagy clears damaged mitochondria, thereby reducing endogenous inflammasome ligands such as mitochondrial reactive oxygen species (mtROS) and mitochondrial DNA (mtDNA), which in turn inhibits inflammasome assembly and activation. Conversely, the NLRP3 inflammasome, by continuously releasing inflammatory mediators during pyroptosis, impairs mitochondrial autophagy, thereby establishing a vicious cycle of “inflammation-mitochondrial damage-inflammation amplification” (Chen et al., 2024). In addition, autophagy not only can limit pyroptosis by eliminating excess mtROS to suppress caspase-11 activation, but also directly modulates the activation, release, and signaling of the downstream pyroptotic mediator IL-1β. (Sun et al., 2017). Studies have demonstrated that, in the setting of MIRI, TSD upregulates autophagy in mouse cardiomyocytes, as evidenced by increased LC3 and decreased p62 levels, thereby inhibiting NLRP3 inflammasome activation and attenuating both pyroptosis and the accompanying inflammatory response (Yang et al., 2024). Benzo [a]pyrene binds to the aryl hydrocarbon receptor (AHR) and activates the p53-BNIP3 signaling axis, which disrupts the Beclin-1–Bcl-2 interaction and consequently blocks autophagy. When autophagic clearance is compromised, NLRP3 accumulates and NLRP3-driven pyroptosis intensifies (Huang et al., 2023). Knockdown of circDGKZ inhibits cardiomyocyte pyroptosis by modulating the miR-345–5p/TLR4/NF-κB axis while concurrently inducing autophagy to accelerate NLRP3 degradation, ultimately ameliorating MIRI (Li S. et al., 2024). Notably, when autophagosomes accumulate beyond a certain threshold through the Beclin-1 pathway, their protective effect is subverted and they instead become a risk factor that promotes cell death. However, the cardioprotective effect of autophagy has an upper limit. When autophagosome accumulation exceeds a critical threshold, autophagy is no longer a protective mechanism but instead becomes detrimental. The cardioprotective effect of empagliflozin is mediated, at least in part, by reducing autophagosome formation (Jiang et al., 2022). This threshold may explain why interventions that merely upregulate autophagy have yielded inconsistent results across different studies; it also suggests that the outcome is determined not by autophagy *per se*, but by the timing and magnitude of autophagic flux activation. Figure 3 summarizes how these mechanisms, the core inflammasome machinery, and the four cardiac cell types converge to place pyroptosis at the center of the MIRI pathological network.

**FIGURE 3:**
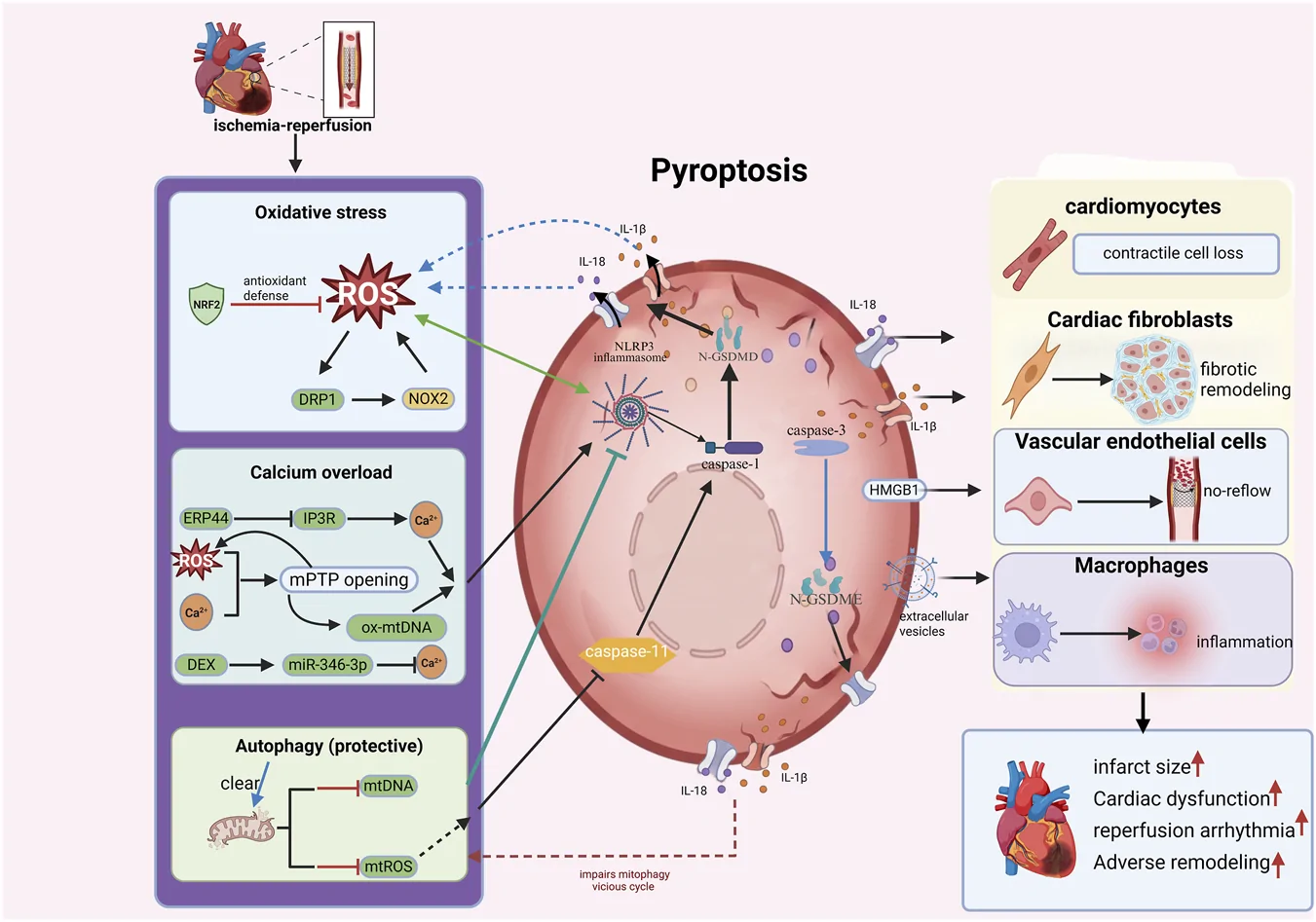
Pyroptosis as a central hub in the pathological network of myocardial ischemia–reperfusion injury. Abbreviations: ROS, reactive oxygen species; DRP1, dynamin-related protein 1; NOX2, NADPH oxidase 2; NRF2, nuclear factor erythroid 2-related factor 2; ERP44, endoplasmic reticulum protein 44; IP3R, inositol 1,4,5-trisphosphate receptor; mPTP, mitochondrial permeability transition pore; ox-mtDNA, oxidized mitochondrial DNA; DEX, dexmedetomidine; mtROS, mitochondrial reactive oxygen species; GSDMD, gasdermin D; N-GSDMD, N-terminal fragment of gasdermin D; N-GSDME, N-terminal fragment of gasdermin E; HMGB1, high-mobility group box 1; IL-1β, interleukin-1β; IL-18, interleukin-18. Created with BioRender.

## Therapeutic strategies targeting pyroptosis in ischemia-reperfusion injury

Based on their targets within the pyroptosis pathway, these therapeutic agents can be classified into NLRP3 inflammasome inhibitors, caspase inhibitors, GSDMD inhibitors, and other categories.

### NLRP3 inhibitors

#### Small-molecule compounds

MCC950 (CRID3) is a highly potent and selective nanomolar-range NLRP3 inhibitor. It blocks ATP hydrolysis by interacting with the Walker B motif within the NACHT domain of NLRP3, thereby inducing a conformational rearrangement that prevents NLRP3 inflammasome assembly and activation (Coll et al., 2019). In addition, MCC950 can inhibit NLRP3 inflammasome activation by blocking a critical step in inflammasome assembly, namely, “ASC oligomerization and speck formation” (Coll et al., 2015). Studies have demonstrated that MCC950 suppresses pyroptosis by inhibiting NLRP3 activation in both *in vitro* and *in vivo* settings, thereby alleviating myocardial ischemia–reperfusion injury (Li Y. et al., 2025). This protective effect has been validated in large animal models: in a porcine myocardial ischemia–reperfusion (I/R) model, the selective NLRP3 inhibitor MCC950 dose-dependently reduced infarct size, improved cardiac function, and attenuated the inflammatory response (Van Hout et al., 2016).

CY-09 directly and selectively inhibits the ATPase activity of NLRP3 by binding to the Walker B ATP-binding motif within its NACHT domain, thereby preventing NLRP3 oligomerization and full inflammasome assembly (Jiang et al., 2017). In a mouse model of MIRI, inhibition of the splenic NLRP3 pathway during the early phase of reperfusion has been shown to attenuate the inflammatory response, reduce infarct size, and improve cardiac function (Xie D. et al., 2023).

INF195 is a novel, synthetically derived small molecule that targets NLRP3. Recent studies have demonstrated that low-dose INF195 blocks NLRP3 oligomerization and assembly by binding to the NACHT domain of the NLRP3 protein, thereby inhibiting the downstream signaling pathway and preventing pyroptosis in cardiomyocytes. INF195 significantly reduces myocardial necrosis and local inflammatory infiltration following myocardial ischemia–reperfusion injury (MIRI), stabilizes cardiac function, and elicits markedly fewer adverse effects than classical NLRP3 inhibitors (Gastaldi et al., 2024).

#### Clinical pharmacotherapy

Metformin is widely prescribed for the treatment of type 2 diabetes and metabolic syndrome owing to its potent insulin-sensitizing effects and favorable safety profile. It also plays a protective role in ischemia–reperfusion injury across multiple organs, including the heart, kidneys, intestine, and brain (Jia et al., 2020; Guo et al., 2023; Wang et al., 2015; Jo et al., 2020). Numerous studies have demonstrated that metformin ameliorates MIRI through diverse mechanisms, such as inhibiting cardiomyocyte apoptosis, attenuating inflammation, alleviating oxidative stress, and modulating autophagy to reduce cell death and improve mitochondrial dysfunction (Shi and Hou, 2025; Fei et al., 2020; Arinno et al., 2021; Osorio-Llanes et al., 2023). A recent study demonstrated that metformin suppresses the NLRP3 inflammasome by activating AMPK signaling. This blockade inhibits cardiomyocyte pyroptosis, thereby alleviating MIRI, improving cardiac function, and attenuating myocardial fibrosis (Zhang et al., 2020). The investigators employed an isolated Langendorff-perfused rat heart MIRI model and neonatal rat ventricular myocytes (NRVMs) subjected to hypoxia/reoxygenation (H/R). Metformin treatment significantly reduced infarct size, inhibited myocardial fibrosis, and downregulated NLRP3 and its downstream proteins. Notably, these protective effects were reversed upon administration of an AMPK inhibitor.

Colchicine is a traditional anti-inflammatory alkaloid originally indicated for the treatment of gout. Accumulating evidence indicates that colchicine confers a degree of cardiovascular protection by blocking NLRP3 inflammasome assembly and suppressing inflammatory cell infiltration (Ding et al., 2026). Microtubule remodeling is essential for NLRP3 inflammasome assembly. Colchicine reversibly binds to β-tubulin and inhibits microtubule polymerization, thereby disrupting the canonical NLRP3 axis. The net effect is attenuated pyroptosis and improved cardiac function (Xia et al., 2026). In the context of MIRI, colchicine alleviates injury primarily by inhibiting pyroptosis through the blockade of NLRP3 inflammasome assembly. In a mouse model of MI, colchicine significantly attenuated NLRP3 inflammasome activation and the expression of downstream pyroptosis-related proteins, and alleviated myocardial injury, inflammation, and fibrosis following MI (Chen Y. et al., 2025). In another animal study, colchicine markedly reduced infarct size 24 h after MIRI in mice, accompanied by significant reductions in troponin T and inflammatory marker levels. At 10 weeks, the colchicine-treated group also exhibited noticeably less myocardial fibrosis (Akodad et al., 2017). Besides animal studies, A randomized controlled trial (RCT) in patients with ST-segment elevation myocardial infarction (STEMI) found no statistically significant difference between treatment groups. Nevertheless, in the colchicine arm, NLRP3 and downstream pyroptosis-related proteins declined steadily from before PCI to 24 h after PCI, whereas in the control arm these markers increased markedly over the same period (Karim et al., 2024).

Glibenclamide is a second-generation sulfonylurea used primarily for the management of type 2 diabetes. It inhibits macrophage pyroptosis by selectively blocking NLRP3 inflammasome activation through the suppression of PAMPs and DAMPs (Lamkanfi et al., 2009). However, glibenclamide inhibits ATP-sensitive K^+^ channels in pancreatic β-cells, thereby promoting massive insulin release, high doses can precipitate life-threatening hypoglycemia. To circumvent this adverse effect, a glibenclamide intermediate (16,673–34–0) was synthetically derived. In a mouse model of MIRI and in corresponding *in vitro* cell experiments, 16,673–34–0 was found not only to significantly reduce infarct size but also to potently and selectively inhibit the NLRP3 inflammasome in both macrophages and cardiomyocytes, thereby blocking the pyroptotic pathway and attenuating inflammatory cardiomyocyte death (Marchetti et al., 2014).

#### Natural products

Aesculin (AES), a hydroxycoumarin glycoside and the active constituent of Fraxinus bark, activates AKT, which in turn phosphorylates GSK3β at Ser9, rendering it inactive. GSK3β inactivation prevents NF-κB p65 phosphorylation and nuclear translocation, thereby suppressing NF-κB-driven NLRP3-mediated pyroptosis, reducing pro-inflammatory cytokine release, attenuating myocardial inflammation, and improving cardiac function (Xu et al., 2021). Stachyose is a water-soluble oligosaccharide present in a broad range of herbs. As a natural prebiotic, it not only modulates the gut microbiota, but also attenuates inflammation, showing therapeutic promise in inflammatory diseases (He et al., 2020). In a mouse model of MIRI, stachyose was found not only to significantly reduce the protein expression of NLRP3, caspase-1, and the N-terminal fragment of GSDMD (GSDMD-N) in cardiac tissue, but also to directly bind NLRP3 to inhibit NLRP3 mediated pyroptosis, thereby conferring a protective effect (Zhang A-Y. et al., 2024). Additional natural products have also been identified that inhibit NLRP3. For instance, curcumin suppresses NLRP3 activation through multiple mechanisms, including blocking ATP-mediated K^+^ efflux, reducing lysosomal rupture, scavenging mitochondrial ROS, and selectively inhibiting the TLR4/NF-κB pathway. It has been shown to attenuate pyroptosis in the MIRI model; similarly, emodin and kaempferol have been demonstrated to inhibit the NLRP3 inflammasome in the I/R model (Kumar et al., 2025).

### Caspase inhibitor

#### Caspase-1 inhibitor

VX-765 is a highly selective caspase-1 inhibitor that is efficiently converted *in vivo* into the active metabolite VRT-043198, which potently inhibits caspase-1, attenuates the release of IL-1β and IL-18, and exerts anti-inflammatory effects across numerous inflammatory models (Wannamaker et al., 2007). In a mouse model of MIRI, administration of VX-765 resulted in a significant reduction in infarct size; when combined with the P2Y12 inhibitor ticagrelor, the reduction in infarct size was greater than that achieved with VX-765 alone (Yang et al., 2017). Another study demonstrated that, in a mouse MIRI model, VX-765 not only reduced infarct size and improved left ventricular systolic function but also markedly lowered the levels of the downstream pyroptotic cytokine IL-1β and suppressed the release of LDH, a marker associated with pyroptosis (Audia et al., 2018). Further experiments revealed that VX-765 inhibits the downstream pathways of pyroptosis exclusively through caspase-1 inhibition, without affecting the upstream signaling events. In a diabetic mouse MIRI model, VX-765 significantly reduced the levels of downstream pyroptosis markers, including cleaved caspase-1, GSDMD, IL-1β, and IL-18, whereas the upstream components NLRP3 and pro-caspase-1 remained essentially unchanged (Zhou et al., 2024). Beyond the canonical pyroptosis pathway, VX-765 also alleviates MIRI through the RISK (PI3K/AKT) pathway, thereby conferring cardioprotection in isolated mouse hearts (Do et al., 2018).

ML132 is a selective, high-potency caspase-1 inhibitor. Its nitrile group forms a covalent yet reversible thioimidate intermediate with the active-site cysteine residue (C285) of caspase-1, thereby blocking enzymatic activity. ML132 has been investigated in ischemic disease, Huntington’s disease, rheumatoid arthritis, inflammatory bowel disease, and sepsis (Boxer et al., 2026). However, studies examining ML132 in the setting of myocardial ischemia–reperfusion injury (MIRI) remain lacking.

#### Other caspase inhibitors

Z- VAD-FMK is a broad-spectrum, irreversible caspase inhibitor that binds to the cysteine residues at the active sites of all caspases, thereby blocking their catalytic function. Its targets include the apoptosis-related caspases 3, 7, 8, and 9, as well as the inflammatory caspases 1, 4, 5, and 11 (Van Noorden, 2001). In a mouse model of myocardial ischemia–reperfusion injury (MIRI), Z-VAD-FMK markedly suppressed the release of the downstream inflammasome effector IL-1β (Sandanger et al., 2013). Z-VAD-FMK has also demonstrated efficacy in other inflammatory conditions. In bronchiectasis, for instance, it potently inhibits neutrophil caspase-1, thereby reducing IL-1β production and suppressing inflammasome-driven inflammation (Chen Z. et al., 2025). Gemfibrozil acts by inhibiting caspase-11, thereby preventing the cleavage of full-length GSDMD into its pore-forming N-terminal fragment. This reduces membrane pore formation, suppresses pyroptosis, and alleviates MIRI (Marunouchi et al., 2025). Telaprevir protects the murine myocardium against ischemia–reperfusion injury in a dose-dependent manner. Its core mechanism involves disrupting the MALT1/TRAF6 signaling axis, which blocks TRAF6-mediated K63-linked polyubiquitination of caspase-11 and consequently suppresses caspase-11-dependent cardiomyocyte pyroptosis (Li M-R. et al., 2025).

#### GSDMD inhibitor

GSDMD is the terminal effector of pyroptosis; inhibiting GSDMD can suppress the release of inflammatory cytokines, rendering it an attractive therapeutic target. Currently, specific inhibitors remain at the stage of basic research and development. Disulfiram (DSF) is an FDA-approved drug for the treatment of chronic alcohol dependence. Recent studies have demonstrated that DSF specifically inhibits GSDMD pore formation by covalently modifying the Cys191 and Cys192 residues in both human and mouse GSDMD, without affecting the upstream proteolytic processing of the protein. By completely blocking GSDMD-mediated membrane pore formation, DSF prevents the release of IL-1β, IL-18, and HMGB1, thereby exerting anti-pyroptotic and anti-inflammatory effects (Hu et al., 2020). Validation studies in primary cardiomyocytes and in both acute and chronic rat models of MIRI have confirmed that the cardioprotective effect of DSF is dependent on the blockade of GSDMD-mediated pyroptosis, independent of its aldehyde dehydrogenase (ALDH)-inhibiting activity. Notably, DSF not only limits acute infarct size but also preserves post-infarct cardiac contractile function over the long term (Chen et al., 2023). Although most earlier studies suggested that DSF acts primarily by inhibiting GSDMD, recent evidence indicates that DSF can also specifically inhibit the NLRP3 inflammasome. Mechanistically, DSF blocks zDHHC5-mediated palmitoylation of NLRP3 at Cys126, thereby preventing NLRP3 localization to the trans-Golgi network (TGN) and ultimately inhibiting inflammasome assembly and pyroptosis (Xu et al., 2024). Whether DSF can alleviate MIRI by directly targeting NLRP3 remains to be tested experimentally. Given the well-documented DSF, its application in the cardiovascular field is severely constrained. By contrast, GI-Y1 is a novel, highly selective GSDMD inhibitor. GI-Y1 targets a single residue, Arg7 of GSDMD, to block GSDMD-N lipid binding and pore formation at both the plasma and mitochondrial membranes, without affecting caspase-1 or caspase-11. In multiple *in vivo* models of MIRI and *in vitro* H/R models, GI-Y1 has been shown not only to suppress GSDMD-mediated pyroptosis and inflammation, but also to prevent GSDMD-N mitochondrial enrichment. This, in turn, restores mitochondrial membrane potential and ATP production, reverses ultrastructural damage, and ultimately limits infarct size, attenuates cardiac fibrosis, and improves cardiac function (Zhong et al., 2023).

#### Other agents

In addition to the agents described above, other compounds that fall outside these categories can also mitigate MIRI by targeting pyroptosis. For instance, Trimetazidine simultaneously inhibits the classical TLR4/MyD88/NF-κB/NLRP3/caspase-1 pathway and the non-canonical caspase-11 pathway, thereby reducing GSDMD cleavage and IL-1β release and alleviating myocardial injury (Chen et al., 2022). Oxytocin activates AMPK signaling, which in turn suppresses NLRP3 inflammasome-mediated cardiomyocyte pyroptosis, consequently attenuating myocardial injury induced by high glucose combined with I/R (Yao et al., 2022). Dexmedetomidine (DEX) downregulates miR-665, thereby relieving its suppression of MEF2D. The resulting upregulation of MEF2D promotes Nrf2 nuclear translocation, which subsequently blocks the pyroptotic pathway and markedly attenuates MIRI (Wang et al., 2023). Hydrogen gas (H_2_) scavenges byproducts of mitochondrial oxidative stress, suppresses the NLRP3 inflammasome, and blocks downstream pyroptosis, thereby reducing hypoxic cardiomyocyte death. In rats, daily inhalation of 2% H_2_ for 3 h over 28 days significantly improved cardiac systolic function and attenuated myocardial fibrosis (Nie et al., 2021b). Rutin suppresses NF-κB pathway activation, downregulates NLRP3 inflammasome expression, and blocks caspase-1/GSDMD-mediated pyroptosis, thereby curbing the release of IL-1β, TNF-α, and IL-18 and alleviating MIRI (Tian et al., 2025).

#### Levels of evidence

The agents discussed above differ markedly in the strength of the evidence behind them, and this should be kept in mind when comparing their reported efficacy. Table 1 summarizes the experimental model and evidence category for each agent. Most compounds have been tested only in cell-based H/R systems and small-animal models; CY-09, INF195, GI-Y1, the glibenclamide intermediate 16,673-34-0, and the natural products belong to this category. MCC950 is the only agent with large-animal validation, having reduced infarct size dose-dependently in a porcine I/R model. Several compounds are clinically approved drugs whose anti-pyroptotic effects have been examined only preclinically, including metformin, glibenclamide, disulfiram, trimetazidine, and dexmedetomidine. Colchicine is notable as the only agent with clinical data in myocardial infarction, a randomized trial in STEMI patients showed no difference was observed between groups in the primary endpoint but demonstrated that Colchicine inhibited the elevation of NLRP3 and its downstream pyroptosis markers after PCI. At the level of the IL-1 pathway more broadly, anakinra has shown signals of benefit in small STEMI trials (Abbate et al., 2010; Abbate et al., 2013), while a trial in non-ST elevation acute coronary syndromes did not (Morton et al., 2015), and canakinumab reduced cardiovascular events in patients after myocardial infarction with residual inflammation in the CANTOS trial (Ridker et al., 2017). The agents discussed above also differ in their scope of action according to the node of the pyroptosis pathway that they target. Upstream inhibitors of NLRP3 or caspase-1 suppress the pathway as a whole, thereby reducing IL-1β/IL-18 maturation, gasdermin cleavage, and pyroptotic cell death, whereas terminal GSDMD inhibitors act at the pore-forming step and more selectively prevent lytic cell death together with the release of intracellular contents. These differences also carry safety implications, because broad suppression of innate immune effectors entails a greater risk of host-defense impairment and infection, as observed in the canakinumab and anakinra trials, whereas GSDMD-targeted agents act more narrowly and may leave upstream inflammatory signaling largely intact, although their efficacy is probably most evident when pyroptotic cell death itself dominates the injury. The translation gap between preclinical efficacy and clinical evidence is therefore considerable, and for most agents the clinical relevance remains unknown.

**TABLE 1:** Common Targeted Inhibitors against pyroptosis for myocardial ischemia-reperfusion injury.

Drug (refs)	Target	Core mechanism → reported effect	Model (Evidence)
MCC950 [115–118]	NLRP3	Blocks NLRP3 ATP hydrolysis (Walker B motif) → ↓infarct size, ↑cardiac function, ↓inflammation	Mouse I/R; porcine I/R (rodent; large animal)
CY-09 [119–120]	NLRP3	Inhibits NLRP3 ATPase → ↓infarct size, ↑cardiac function	Mouse I/R (rodent)
INF195 (Gastaldi et al., 2024)	NLRP3	Blocks NACHT-domain oligomerization, fewer adverse effects → ↓necrosis, stabilized function	Mouse I/R (rodent)
Metformin (Zhang et al., 2020)	NLRP3	AMPK activation suppresses NLRP3 → ↓infarct, fibrosis, pyroptosis markers	Langendorff rat heart I/R; NRVM H/R (rodent; *in vitro*)
Colchicine [132–135]	NLRP3	Blocks microtubule-dependent assembly → ↓infarct/fibrosis (mouse); ↓NLRP3 markers after PCI (human, no clinical difference)	Mouse MI; STEMI patients undergoing PPCI (rodent; human RCT)
Glibenclamide (Lamkanfi et al., 2009)	NLRP3	Suppresses PAMP/DAMP-triggered activation → ↓macrophage pyroptosis	Macrophage models (*in vitro*)
5-chloro-2-methoxy-N-[2-(4-sulfamoylphenyl)ethyl]benzamide (Marchetti et al., 2014)	NLRP3	Same mechanism with Glibenclamide, no hypoglycaemic risk → ↓infarct size	Mouse I/R; NRVM H/R (LPS + ATP) (rodent; *in vitro*)
Aesculin (Xu et al., 2021)	NLRP3	Blocks NF-κB p65 nuclear translocation → ↓inflammation, ↑cardiac function	Mouse I/R (rodent)
VX-765 [141–145]	Caspase-1	Selective caspase-1 inhibition (VRT-043198) → ↓infarct size, ↑LV function, ↓IL-1β/LDH; RISK activation	Mouse I/R (incl. diabetic); isolated rat heart (rodent)
Disulfiram [151–152]	GSDMD	Modifies Cys191/192, blocks pore formation → ↓acute infarct, preserves long-term function	Primary cardiomyocytes; acute and chronic rat MIRI (rodent; *in vitro*)
Trimetazidine (Chen et al., 2022)	multi-target	simultaneously inhibits the canonical TLR4/MyD88/NF-κB/NLRP3/caspase-1 axis and the non-canonical caspase-11 pathway	Mouse I/R (rodent)
Dexmedetomidine (Wang et al., 2023)	multi-target	Nrf2 nuclear translocation blocks pyroptosis → ↓infarct size	Mouse I/R (rodent)

Table legend: Summary of small-molecule drugs, clinical agents and natural products that suppress pyroptosis in MIRI models, including their direct molecular targets, core anti-pyroptotic mechanisms and corresponding cited references.

#### Translational barriers

Some barriers stand between these preclinical findings and clinical use. The first is timing. Most studies administer the agent before ischemia or at the onset of reperfusion, a schedule that is rarely feasible in patients, in whom the diagnosis is usually made after reperfusion has begun. VX-765 administered at reperfusion and colchicine given before PCI are the exceptions that match clinical reality, and future studies should adopt post-reperfusion treatment schedules as the default. The second barrier is specificity and safety. NLRP3 and caspase-1 are components of host defense, and their systemic inhibition carries an infection risk. In the CANTOS trial, canakinumab increased the rate of fatal infection despite its cardiovascular benefit (Ridker et al., 2017), and the MRC-ILA trial of anakinra showed no benefit and more infections (Morton et al., 2015). Agents with fewer adverse effects, such as INF195, or with a more favorable safety profile, such as the glibenclamide intermediate that avoids hypoglycemia, are therefore attractive, but their selectivity *in vivo* remains to be established. The third barrier is combination therapy. Patients with STEMI routinely receive antiplatelet and anticoagulant therapy, yet only one study, combining VX-765 with the P2Y12 inhibitor ticagrelor, has examined the interaction between pyroptosis inhibition and antithrombotic treatment, and it reported additive infarct reduction, but the safety of this combined therapy was not assessed. The fourth barrier is endpoint selection. Infarct size is the most common readout, but it is measured by different methods at different time points across studies, and the colchicine trial illustrates the disconnect between biomarker changes and clinical outcome. Whether pyroptosis-targeted therapy can improve hard clinical endpoints, or whether it should be positioned as an adjunct to reduce reperfusion injury and remodeling remains an open question. For the clinically approved drugs discussed above, dose selection is a further complication, because anti-inflammatory doses may differ from the doses used for their original indications; colchicine, for example, is used at much lower doses in cardiovascular disease than in gout.

## Future directions and outlook

The canonical NLRP3/caspase-1/GSDMD axis has been the most thoroughly characterized. As research has progressed, GSDME/caspase-3 has been found to be increasingly associated with the cardiovascular system. For example, GSDME has been detected in various cell types within atherosclerotic plaques, including endothelial cells (ECs), macrophages, and vascular smooth muscle cells. In ECs, GSDME damages mitochondria, leading to the release of mitochondrial DNA (mtDNA), which activates the cGAS–STING pathway and thereby promotes vascular inflammation (Xie S. et al., 2023). In macrophages, GSDME is upregulated by oxidized LDL (ox-LDL) in advanced atherosclerotic lesions; it also enhances caspase-3 activity, shifting the mode of cell death from apoptosis to pyroptosis; the resulting macrophage pyroptosis accelerates plaque progression (Wei et al., 2023). In VSMCs, GSDME-mediated pyroptosis is a key driver of coronary artery calcification. In pulmonary arterial hypertension (PAH), silencing of GSDME expression significantly reduced ventricular systolic pressure and attenuated right ventricular hypertrophy (RVH). Furthermore, GSDME is also implicated in non-ischemic cardiomyopathies, including dilated cardiomyopathy, hypertrophic cardiomyopathy, diabetic cardiomyopathy, doxorubicin (DOX)-induced cardiomyopathy, and septic cardiomyopathy (Yang et al., 2026). To date, GSDMD is the most extensively studied member of the gasdermin family in the context of MIRI. The other members—GSDMA, GSDMB, GSDMC, and GSDME—have been more thoroughly investigated in other cardiovascular diseases and in tumors, with particular emphasis on GSDME. Future studies clarifying the specific roles and dynamic changes of GSDME at different stages of MIRI may facilitate the clinical translation of therapeutic strategies that target its upstream or downstream pathways, which could be of critical importance for the treatment of MI and its complications.

The current bottleneck in this field is no longer a shortage of candidate compounds, but rather the elucidation of key scientific questions. Although dozens of pyroptosis inhibitors have been reported, the specific cell types undergoing pyroptosis in the reperfused heart, as well as the temporal pattern and spatial localization of pyroptosis, remain to be precisely determined. Nearly all conclusions concerning the canonical NLRP3/caspase-1/GSDMD signaling axis have been derived from studies of whole-tissue (bulk) samples from mice. The human MI atlas generated by Kuppe et al. was the first to apply single-nucleus RNA sequencing (snRNA-seq) and spatial transcriptomics to this field (Kupp et al., 2022), combined with GSDMD/GSDME reporter mouse models, this approach could advance the generalized conclusion that pyroptosis occurs during MIRI toward a cell-type–specific statement with well-defined spatiotemporal characteristics. The other major limitation concerns the experimental models. Most existing studies are based on the H9c2 cardiomyocyte cell line and neonatal rat cardiomyocytes, which differ considerably from the clinical pathology. Human induced pluripotent stem cell (hiPSC)-derived cardiac organoids and heart-on-a-chip systems enable H/R studies at the human multicellular level; organoids derived from diabetic or aged donors could directly test whether the comorbidity-dependent effects observed in mouse MIRI also manifest within the human genetic background. Given the breadth of preclinical evidence reviewed here, we are cautiously optimistic that pyroptosis inhibition will join the cardioprotective armamentarium; the next 5 years should determine whether this optimism is justified.

## Conclusion

Pyroptosis couples cell death to the inflammatory cascade and pervades the entire pathological course of MIRI. Spanning inflammasome assembly, gasdermin-mediated membrane perforation, single-cell injury, and multicellular crosstalk that amplifies the inflammatory response, the pyroptosis axis constitutes a central regulatory hub within the MIRI pathological network. Targeting pyroptosis and translating diverse therapeutic strategies that intervene at its upstream or downstream pathways into clinical practice is therefore essential for advancing the treatment of MI and its associated complications.
